# The Anatomy, Histology, and Function of the Major Pelvic Ganglion

**DOI:** 10.3390/ani14172570

**Published:** 2024-09-04

**Authors:** Jessica Natalia Landa-García, María de la Paz Palacios-Arellano, Miguel Angel Morales, Gonzalo Emiliano Aranda-Abreu, Fausto Rojas-Durán, Deissy Herrera-Covarrubias, María Rebeca Toledo-Cárdenas, Jorge Manuel Suárez-Medellín, Genaro Alfonso Coria-Avila, Jorge Manzo, Maria Elena Hernández-Aguilar

**Affiliations:** 1 Doctorado en Investigaciones Cerebrales, Universidad Veracruzana, Xalapa 91190, Veracruz, Mexico; natalialanda15@gmail.com (J.N.L.-G.); arellanop.maripaz960@gmail.com (M.d.l.P.P.-A.); 2 Instituto de Investigaciones Biomédicas, Universidad Nacional Autónoma de México, Mexico City 04510, Mexico; mamm@iibiomedicas.unam.mx; 3 Instituto de Investigaciones Cerebrales, Universidad Veracruzana, Xalapa 91190, Veracruz, Mexico; garanda@uv.mx (G.E.A.-A.); frojas@uv.mx (F.R.-D.); dherrera@uv.mx (D.H.-C.); rtoledo@uv.mx (M.R.T.-C.); josuarez@uv.mx (J.M.S.-M.); gcoria@uv.mx (G.A.C.-A.); jmanzo@uv.mx (J.M.)

**Keywords:** major pelvic ganglion, pelvic area, parasympathetic and sympathetic innervation, rat, cat, dog, pig

## Abstract

**Simple Summary:**

In male rats, the major pelvic ganglion is the principal component of the pelvic plexus and plays a crucial role in regulating various physiological functions such as urination, defecation, erection, ejaculation, and glandular secretion. This ganglion is considered mixed, as it receives both sympathetic and parasympathetic innervation through the hypogastric and pelvic nerves, respectively. Homologous structures with similar functions are present in other species, including cats, dogs, and pigs; however, differences exist in nomenclature, anatomical complexity, and functionality. Although anatomical, histological, and immunohistochemical studies have been conducted on these structures across various species, the major pelvic ganglion of the rat has been the most extensively studied due to its ease of identification and manipulation.

**Abstract:**

This review provides a comprehensive analysis of the pelvic plexus and its regulation across various mammalian species, including rats, cats, dogs, and pigs. The pelvic and hypogastric nerves play crucial roles in regulating pelvic functions such as micturition, defecation, and erection. The anatomical organization of these nerves varies, forming either well-defined ganglia or complex plexuses. Despite these variations, the neurons within these structures are consistently regulated by key neurotransmitters, norepinephrine and acetylcholine. These neurons also possess receptors for testosterone and prolactin, particularly in rats, indicating the significant role of these hormones in neuronal function and development. Moreover, neuropeptides such as vasoactive intestinal peptide (VIP), substance P, neuropeptide Y (NPY), somatostatin (SOM), galanin (GAL), and calcitonin gene-related peptide (CGRP) are co-released with neurotransmitters to modulate pelvic functions. This review highlights the complex interplay between neurotransmitters, neuropeptides, and hormones in regulating pelvic physiology and emphasizes the importance of hormonal regulation in maintaining the functionality and health of the pelvic plexus across different species.

## 1. Introduction

The major pelvic ganglion (MPG) is a key structure within the pelvic plexus, a complex network of nerve fibers and neuronal bodies strategically located among the pelvic organs. This ganglion plays a critical role in regulating various physiological functions, including urination, defecation, erection, ejaculation, and glandular secretion, by transmitting nerve signals from the central nervous system to the pelvic organs, such as the urinary tract, lower intestine, and reproductive organs [1,2,3].

In male rats, the MPG constitutes the principal component of the pelvic plexus, complemented by accessory ganglia, also known as peripheral ganglia. The nomenclature and function of these homologous structures may vary among different mammalian species. For instance, in females, it is referred to as the paracervical ganglion or Frankenhauser’s ganglion, while in rabbits and guinea pigs, the term “pelvic plexus” is used due to its anatomical complexity. In cats, this structure is known as the “inferior hypogastric plexus,” and in dogs, it is termed the “periprostatic plexus,” reflecting anatomical and functional differences between species [2,3,4,5].

Therefore, this review aims to compare the anatomical, histological, and functional characteristics of the major pelvic ganglion across various mammalian species, including dogs, guinea pigs, cats, pigs, and male rats. Aspects such as anatomy, nerve connections, histology, neurochemistry, and physiology will be addressed to enhance the understanding of similarities and differences in pelvic function regulation among these species.

### 1.1. General Overview and Anatomical Organization

**Figure 1:**
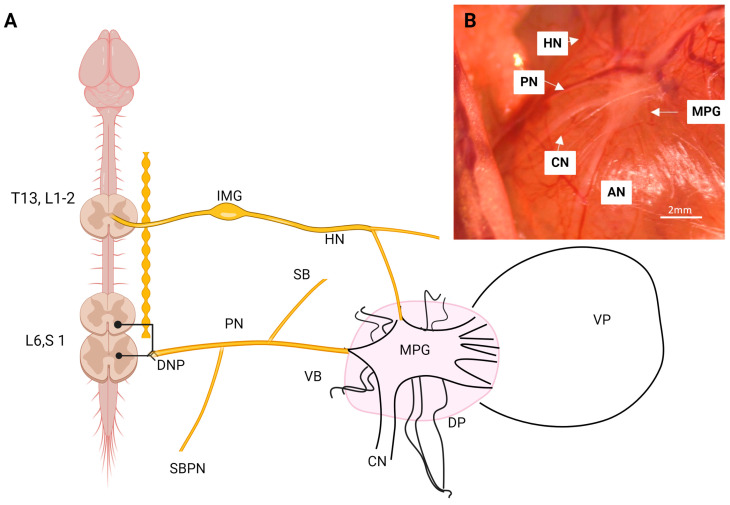
Schematic representation of the pelvic plexus. (**A**) Pelvic plexus of the male rat. (**B**) Photomicrograph of the right pelvic ganglion of the adult male rat. VP, ventral prostate; AN, accessory nerves; DP, dorsal prostate; CN, cavernous nerve; MPG, major pelvic ganglion; PN, pelvic nerve; HN, hypogastric nerve; DNP, dorsal nerve of the penis; IMG, inferior mesenteric ganglia; VB, viscerocutaneous branch of the pelvic nerve; SBPN, sensory branch of pudendal nerve; VP, ventral prostate; DP, dorsal prostate.

The MPG, also known as the inferior hypogastric ganglion, is a crucial structure in the autonomic nervous system of mammals. It is part of the pelvic plexus and plays a vital role in regulating the visceral functions of the pelvic region, including the bladder, rectum, and reproductive organs. It is in the lower part of the pelvic cavity, adjacent to the hypogastric arteries (also known as internal iliac arteries) and near the rectum and bladder. It is bilaterally symmetrical, attached to the serous cavity of the dorsal lobe of the prostate, and connected by ipsilateral and contralateral commissural nerves. The ganglion measures approximately 2 × 4 mm (Figure 1), has a pyramidal shape, and exhibits characteristics of sexual dimorphism [6,7,8,9,10,11,12,13,14].

The pelvic plexus in male rats comprises three pairs of accessory ganglia (AG) and one pair of major pelvic ganglia (MPG). The AG, also known as peripheral ganglia or hypogastric ganglia, are located near the base of the deferent ducts. Historically, they have been considered identical to the MPG, although with lesser innervation to certain reproductive organs. These ganglia are situated close to the midline and communicate with the larger ganglia located laterally on the opposite side (Figure 1A), allowing preganglionic and postganglionic axons to innervate contralateral neural structures [13].

The pelvic plexus varies among species. For example, in guinea pigs, there are an anterior major pelvic ganglion (AMPG), located at the entrance of the deferent ducts into the bladder, which resembles the MPG of the male rat, and a posterior major pelvic ganglion (PMPG). These ganglia are responsible for innervating the urinary bladder, the distal portion of the urethra, the deferent ducts, the seminal vesicle, a portion of the prostate, the penis, and the rectum [15,16,17,18,19,20,21]. In pigs, the pelvic plexus is composed of a network of filaments and several small ganglia surrounded by connective tissue, located in the medial part of the iliac vessels and extending bilaterally to the lower part of the peritoneum and the lobulated external surface of the seminal vesicles [22]. They also have an AMPG located between the proximal end of the deferent duct and the caudal part of the seminal vesicle, in addition to the PMPG. In contrast, cats have a more complex pelvic plexus, where the pelvic nerves converge and intertwine with the ganglionic plexus at the base of the bladder, forming fine filaments that extend to the lateral external surface of the detrusor muscle. This structure comprises between 40 and 60 ganglia, each containing approximately 10 to 20 neurons with extensive dendritic branching [1,3,23]. In dogs, the pelvic plexus (PP) is located 5 to 10 mm lateral to the prostate, just beneath the parietal pelvic peritoneum and ventrolateral to the rectum. It typically appears as a well-defined, rhomboid, and flat structure, with dimensions ranging from 5 to 15 mm [5].

Although there are notable differences in the anatomy of pelvic ganglia among laboratory animals, the physiological importance, advantages, and disadvantages of neuronal aggregation in these ganglia, as opposed to dispersion in a large plexus, have not yet been reported. This underscores the importance of understanding anatomical differences among species for clinical and research applications.

Regarding its organization, in most mammals, this ganglion consists of sympathetic and parasympathetic neurons that synthesize adrenaline or acetylcholine, respectively. The acetylcholine-synthesizing neurons are divided into two types, differing in size and staining intensity. The most prevalent cell type in the ganglion ranges from 30 to 45 μm in size and shows weak to moderate immunostaining. The other cell type measures between 15 and 25 μm and exhibits very intense immunostaining. These neurons are located at the elongated end of the ganglion, mainly grouped at the dorsal end of the pelvic ganglion and around the pelvic nerve as it enters the major pelvic ganglion [24]. Additionally, the ganglion contains a large population of small, intensely fluorescent cells (SIF) with coarse granules in their cytoplasm, and many ganglion cells are closely juxtaposed by SIF cells [24]. In rats, two types of SIF cells have been identified based on the size and morphology of their granules. The most prevalent SIF cells contain numerous vesicles with diameters ranging from 1500 to 2700 Å. The second type of SIF cell is characterized by small vesicles, between 500 and 1200 Å, located peripherally. These cells can receive afferent innervation from a ganglion cell but also innervate ganglion cells, possibly contacting satellite cells responsible for generating a regulatory microenvironment. It has been reported that in this type of efferent innervation, the nerve fibers contain numerous smaller clear vesicles compared to those observed in SIF cells. Furthermore, SIF cells may appear singly or in groups of varying sizes, dispersed among both cholinergic and adrenergic ganglion neurons. Although much is still unknown about the function of these cells in the ganglion, it is suggested that they contain norepinephrine, without ruling out the possibility of synthesizing other neurotransmitters such as catecholamines. A notable feature of these cells is their proximity to blood vessels, which allows them to be hormonally modulated [24,25].

Within the ganglion, as in all autonomic ganglia, there are also satellite cells characterized by relatively simple morphology. These cells typically ensheathe neuron cell bodies, are flat, and contain little cytoplasm. As they encase the ganglion cell, it is proposed that they protect the neuron from exposure to molecules from the circulation, modulating neuronal function by allowing the passage of some proteins and macrophages, although this is not yet fully understood. Structurally, it has been observed that the surface in contact with the connective tissue is flatter compared to the internal surface, increasing the contact area with the neuron [26]. The neuron–satellite cell unit is commonly observed, but there may be clusters where a single satellite cell surrounds two or three neurons [27]. It is also possible for a neuron to be surrounded by several satellite cells, depending on the neuron’s size. For example, in geckos, the ratio is 3.8:1; in mice, it is 5.5:1; in rats and mice, it is 8.2:1; and in rabbits, it is 10.2:1 [28]. Characteristic of satellite cells is that they form a functional syncytium because they are interconnected by gap junctions assembled by the Cx43 protein, and they have immune properties as they express proteins such as CD40, 54, 14, and 68 [29,30,31].

The other cell type present in the ganglion is Schwann cells, the most abundant glial cells in the peripheral nervous system. Their primary function is to cover the axon with myelin sheaths composed of 70–85% lipids and 15–30% proteins, increasing the conduction velocity of nerve impulses through saltatory conduction. These cells influence axon structure and diameter and protect the axon from mechanical damage. Additionally, they can modulate synaptic activity and play a key role in synaptic competition [32,33,34,35].

**Figure 2:**
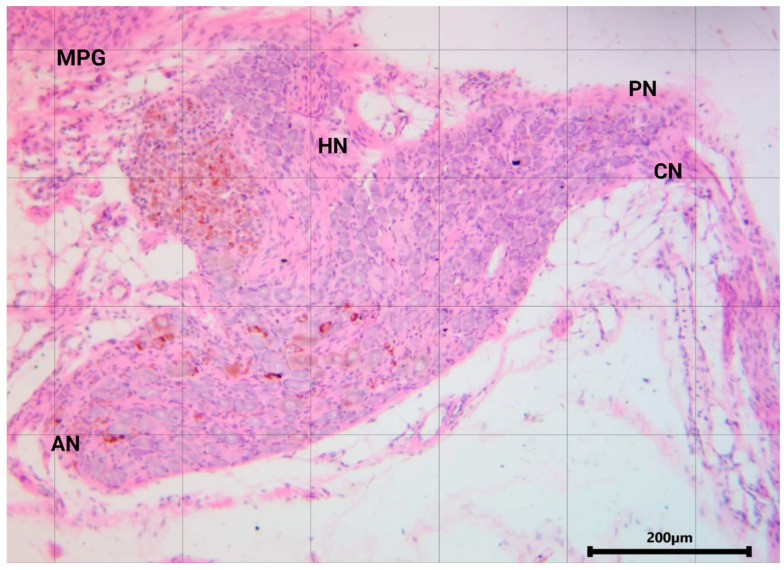
Hematoxylin and eosin staining of the MPG in the male rat. AN, accessory nerves; CN, cavernous nerve; PN, pelvic nerve; HN, hypogastric nerve.

In summary, this ganglion shares characteristics with other autonomic ganglia in terms of cell types. However, its distinction lies in being a mixed ganglion, as it contains both sympathetic and parasympathetic fibers from the hypogastric and pelvic nerves (Figure 2).

### 1.2. Preganglionic and Postganglionic Connections

MPG function is regulated by preganglionic fibers originating from the hypogastric nerve (HN), typically classified as sympathetic, and the pelvic nerve (PN), which is parasympathetic. This dual innervation classifies the ganglion as mixed, as it contains both sympathetic and parasympathetic fibers [36,37,38,39].

Sympathetic fibers originate from the intermediolateral cell column of thoracolumbar segments T10-L2. The axons of these neurons form the lumbar splanchnic nerves (LSN), which project to the inferior mesenteric ganglion (IMG), as passage fibers without making synapses. From the caudal area of the IMG, the HN, comprised of these preganglionic axons, descends, joining the inferior hypogastric plexus, travels behind the ureter, and branches into hypogastric accessory nerves and the main HN, which terminates in the rostral region of the major pelvic ganglion [7,40,41,42]. In rats, the preganglionic fibers composing this nerve originate from 81% of neurons located in the dorsal gray commissure of the L1–L2 spinal segments, with diameters between 24 and 21 µm, respectively [43]. This nerve comprises 223 unmyelinated fibers and 1363 myelinated fibers, constituting 85% of the total fibers present in the nerve. Few afferent or sensory fibers are present, as 96% of these fibers remain in the nerve following dorsal root ganglectomy and 65% remain after ventral rhizotomy. In cats, these neurons are in segments L2–L5, predominantly in segments L3–L5 in the intermediolateral horn, where preganglionic sympathetic neurons are found. The number of neurons composing this nerve is approximately 20,000, with cell bodies diameters of 300–400 µm, closely approximating the 21,000 fibers in the composite nerve, which includes 1300 afferent fibers (6.5%), 1700 preganglionic fibers (8.5%), and the remainder as postganglionic fibers (85%) [44]. Compared to the total fibers in rats, this represents a 13-fold increase [45]. Thus, nerves emerging from the major pelvic ganglion and directed toward the ventral lobe originate from the ventral area, while those innervating the dorsolateral lobe originate from the rostral-dorsal area of the major pelvic ganglion [3,46].

In rats, preganglionic neurons originating from the pelvic nerve are in the intermediolateral gray substance of the L6-S1 spinal segments [47,48]. Approximately 550 neurons, measuring 10 × 20 µm, were in the intermediolateral gray with dendrites extending toward the lateral follicle, and an average of 1500 sensory neurons were also reported [49]. In monkeys, an average of 820 preganglionic neurons are found in the sacral parasympathetic nucleus in the intermediate gray of S1–S3 segments, a pattern similar to that found in cats [50]. Regardless of the spinal segments involved, it is evident that the sacral parasympathetic system exhibits a similar organization across various mammalian species [48,51]. In rats, nerve fibers emerge from the ventral horn and branch into the somatomotor branch, innervating the iliococcygeus and pubococcygeus muscles, and the viscerocutaneous branch, which enters the dorsal region of the major pelvic ganglion [47]. It is important to note that information exiting these nerve terminals is regulated by interneurons in contact with the neurons forming this nerve. Thus, preganglionic neurons receive excitatory glutamatergic inputs from two distinct populations of interneurons located in the dorsal and medial regions of the sacral parasympathetic nucleus [52,53]. This partially explains why this nerve, along with the hypogastric nerve, can regulate various functions of the pelvic area.

As previously mentioned, the major pelvic ganglion regulates different pelvic functions through postganglionic nerve fibers emerging from it. In dogs, a prominent efferent branch emerges from the caudal end of the plexus and extends toward the prostatic apex as the main cavernous nerve (CN). After traveling 15 to 20 mm, it branches to send a small branch to the rectum, while the main trunk continues to the prostate apex and along the urethra to the pelvic floor. This main CN measures up to 5 mm in width and 40 to 60 mm in length. In some cases, an additional efferent nerve emerges from the medial edge of the plexus and extends toward the middle prostate, branching widely into the periprostatic fascia and extending to the posterolateral and lateral parts of the prostate, eventually connecting with the main CN [5]. Additionally, this nerve also innervates the bladder, urethra, and ureters along with the hypogastric ganglion [11,54,55,56].

In pigs, the hypogastric nerve, derived from the caudal mesenteric ganglion, descends from the posterior pole of this ganglion on both sides and, as it progresses, runs parallel to the lumbar column before entering the pelvic cavity and joining the pelvic plexus. Unlike in humans, in pigs, this nerve rarely forms an elongated plexus, but appears as long branches that anastomose without forming a compact structure, as observed in rats. This nerve consists of four to ten bundles with a diameter ranging from 22 to 320 μm and four to ten nerve bundles with few myelinated fibers, measuring 1 to 2–3 μm in diameter, distributed irregularly or forming clusters among unmyelinated fibers. On the other hand, pelvic nerves in pigs are thinner than the hypogastric nerve and originate from branches emerging from the S1 segment immediately after exiting the sacral foramen [22]. The pelvic nerve in pigs has a single branch composed of six–twelve nerve bundles, each with a diameter ranging from 30 to 360 μm, wrapped in loose connective tissue. These nerves contain myelinated fibers ranging from 1.7 to 27 μm in diameter [22]. Of the nerves emerging from the pelvic plexus and targeting the pelvic organs in pigs, the pudendal nerve descends along the external surface of the ligament to re-enter the pelvis through the lesser sciatic foramen, receiving a branch from the sciatic nerve upon entry, usually just after exiting the lesser sciatic foramen. This nerve sends branches to the border between the rectum and the bulbourethral gland, one of which extends forward to innervate the bulbourethral gland and the urethra. This branch divides into thinner branches, accompanied by blood vessels, penetrating the urethral muscular wall [22]. The myelinated nerve fibers originating from the pudendal nerve form nerve bundles approximately 150–180 μm in diameter. Within these bundles, nerve fibers exhibit diameters ranging from 5 to 5.5 μm, with some between 8.5 and 13.5 μm [22,57,58,59,60].

In guinea pigs, there are two mesenteric ganglia, anterior and posterior to the inferior mesenteric artery, approximately 3 mm from the aorta. These ganglia are connected by fine nerves, which in turn receive nerve fibers from the third, fourth, and fifth lumbar ganglia of the ganglionic chain [15], directing fibers toward the inferior mesenteric ganglion from which the colonic and hypogastric nerves emerge. The colonic nerves consist of a dozen strands accompanying the inferior mesenteric artery toward the colon, eventually innervating the rectum. There are two main hypogastric nerves and one accessory hypogastric nerve emerging from the inferior mesenteric ganglia. The main hypogastric nerves extend left and right to terminate in the deferent ducts and seminal vesicles [15]. These ganglia, also known as anterior pelvic ganglia, receive connections from pelvic nerves and send fibers to the deferent ducts, seminal vesicles, bladder, prostate, and ureters in males. Other dispersed cell groups along the main hypogastric nerves are considered additional hypogastric ganglia. The posterior pelvic nerves, located deeper in the pelvic cavity, form a plexus connected to the anterior ganglia, contributing to the innervation of organs such as the rectum and urethra. These nerves, primarily originating from the second, third, and fourth sacral nerves, also give rise to branches innervating the perineal and anal areas. Additionally, there are two pudendal nerves, which provide innervation to structures such as the urethra, penis, anal sphincter, and perineal skin [15]. Notably, neuronal activity from the urethra, rectum, and genitals ascends to the spinal cord via the pelvic nerve and is subsequently transmitted through the spinal cord to the cerebral cortex in both dogs and cats [11,61]. Furthermore, the control of seminal emission is controlled by two sympathetic pathways: one originating from the hypogastric nerve and the other from the lumbosacral sympathetic trunks, which appears to function as a compensatory system for seminal emission in the event of hypogastric nerve damage [62,63,64]. In the guinea pig pelvic ganglion, the enzymes tyrosine hydroxylase (TH) and dopamine β-hydroxylase (DBH) are present, along with the enzyme for acetylcholine synthesis, choline acetyltransferase (ChAT). Additionally, two main types of neurons have been reported: a) those containing NOS located in the caudal part of the major pelvic ganglion (APG) and b) TH-positive neurons lacking NOS located in the cranial part [18].

### 1.3. Neurotransmitters and Receptors in the Pelvic Plexus

**Figure 3:**
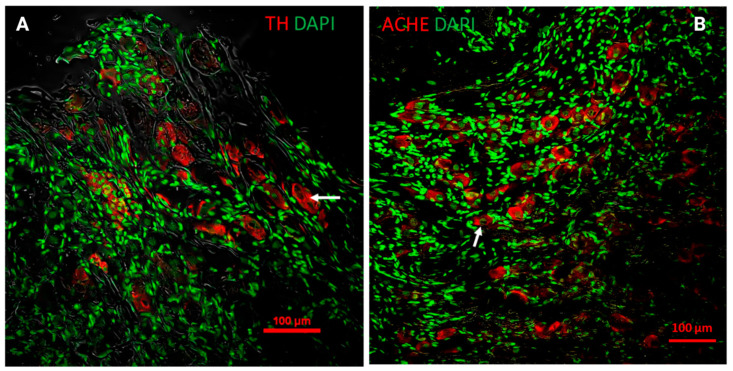
The presence of adrenergic and cholinergic neurons in the MPG of an adult male rat. (**A**) Immunofluorescence for tyrosine hydroxylase (TH). (**B**) Immunofluorescence for acetylcholinesterase (ACHE). Immunostaining (in red) shows the larger size of adrenergic neurons (white arrows). The abundant small green labels correspond to nuclei of satellite cells.

A notable feature of the MPG, at least in rats, is its mixed nature; it contains both sympathetic and parasympathetic fibers, with neurons synthesizing either norepinephrine (adrenergic neurons) or acetylcholine (cholinergic neurons) (Figure 3A,B). It has been argued that all neurons in the MPG are either cholinergic or adrenergic, and they do not synthesize both neurotransmitters, with fewer than 5% of neurons lacking either neurotransmitter [10,39].

These neurons exhibit distinct topography within the MPG. Adrenergic neurons, with areas ranging from 600 to 1400 μm^2^, are located preferentially in the ventral region, near the entry point of the hypogastric nerve (HN). Only a few are found where the HN integrates with the PN (dorsal region), and none are present where the cavernous nerve originates [10,24,65,66]. Sympathetic neurons have been reported to express neuropeptide Y (NPY), which is involved in the norepinephrine-mediated vasoconstriction of pelvic organs [67].

In contrast, cholinergic neurons range from 300 to 700 μm^2^. Dail, Evan, and Eason [24] reported that there is a cluster of small cholinergic neurons that show intense acetylcholine staining and are located in the dorsal region, near the PN, while another population of small neurons with weak acetylcholine staining is found near small branches from the HN [3,24] (see Figure 3B). Additionally, cholinergic neurons co-express neuropeptides, such as somatostatin (SOM), cholecystokinin (CCK), galanin (GAL), calcitonin gene-related peptide (CGRP), neuropeptide Y (NPY), and vasoactive intestinal peptide (VIP), and also express the cocaine- and amphetamine-regulated transcript (CART) peptide [3,24]. It is important to note that afferent fibers innervating the colon are polymodal, responding to temperature and chemical changes [68].

The relative distribution of sympathetic and parasympathetic neurons in the pelvic ganglia varies across species, gender, and strain, highlighting interspecies variations in the neurochemical regulation of pelvic organ function, which may have significant implications for physiology and pathology in these animals.

In the guinea pig’s pelvic ganglion, particularly in the caudal region near the rectal and penile nerves, primarily NOS-immunoreactive neurons are found, and almost all neurons contain choline acetyltransferase (ChAT), indicating cholinergic neurons that are also immunoreactive to neuropeptides such as VIP, NPY, SOM, or dopamine β-hydroxylase (DBH). Conversely, in the cranial region, neurons are abundant in TH-immunoreactivity, suggesting adrenergic neurons, with almost all co-expressing NPY [18]. In the pig’s pelvic ganglion, 40% of the neurons show immunoreactivity to acetylcholine (ChAT) or the vesicular acetylcholine transporter (VAChT), and 39% also exhibit immunoreactivity to NOS, VIP, NPY, or SOM, suggesting the co-expression of neurotransmitters and peptides, highlighting the functional and neurochemical diversity of neurons in the pelvic ganglion [17,69,70]. In dogs, it has been reported that penile erection and bladder control mediated by the pelvic nerve are likely influenced by vasoactive intestinal peptide [61,64,71,72].

### 1.4. Hormonal Regulation in the Pelvic Ganglia

The anatomical and functional organization of the major pelvic ganglion (MPG) and its neurotransmitters and neurohormones that regulate various pelvic functions across different animal species are well understood. Given that reproduction is one of these functions, it is reasonable to investigate whether the development of this ganglion is subject to hormonal regulation, particularly by steroid hormones, with androgens being of primary interest.

Steroid hormones play a crucial role in the development and function of the autonomic nervous system, including the MPG, which is present in various mammalian species. The MPG is a key structure in the innervation of pelvic organs and is influenced by several steroid hormones, especially sex hormones such as androgens and estrogens. In rats, for example, it has been shown that the MPG is sensitive to androgens. Castration performed at twelve hours postnatally affects the activity of enzymes responsible for synthesizing acetylcholine and norepinephrine, an effect that is reversed by administering testosterone within ten days post-castration. Estrogens can also reverse the effects of castration on acetylcholine transferase activity but do not affect tyrosine hydroxylase activity. This study indicates a critical postnatal period dependent on the presence of androgens [73,74,75,76].

Furthermore, it is known that neurons in the MPG are affected by the absence of androgens, as maintained castration for two to seven weeks results in adrenergic neurons reaching only about 60% of their normal size, without affecting cholinergic neurons. This suggests that adrenergic neurons are more sensitive to androgen absence and that ganglion development depends on the presence of this androgen [77]. In vitro studies have revealed that both androgens and estrogens, derived from testosterone due to the presence of aromatase, can stimulate the growth of more complex neurites in both adrenergic and cholinergic neurons expressing nitric oxide synthase (NOS) [78,79]. It is noteworthy that approximately 40% of neurons innervating the penile corpus cavernosum contain androgen receptors; however, only 20% of neurons expressing nitric oxide and 40% of those expressing vasoactive intestinal peptide (VIP) contain androgen receptors [78]. Although the precise mechanisms are not fully understood, it is known that testosterone modifies the membrane potential of these ganglionic neurons by inducing significant depolarizations relative to their resting potential [80], reinforcing the notion that androgen regulation affects the electrophysiological properties of these neurons [81,82].

Considering that the MPG controls reproductive functions and is sensitive to testosterone, it has also been shown that sexual behavior impacts not only the prostate, causing plastic changes in the gland [83], but also affects the ganglion. Experimental conditions reveal that the ganglion area in sexually experienced subjects is larger compared to naïve subjects, likely due to an increase in neuronal area from 0–300 μm^2^ to 600–900 μm^2^. However, when preganglionic innervation is interrupted, these effects are not easily reversed; although there is a slight reduction in neuronal area and size, the effects are not as drastic as in sexually inexperienced animals. This plasticity observed in the MPG appears to be a consequence of elevated blood levels of testosterone and prolactin, both of which have receptors in this ganglion. However, the effects of testosterone and prolactin do not modify their receptor expression nor ganglionic α1-adrenergic and M3 muscarinic receptors [37,84]. Conversely, the absence of preganglionic neural control or axotomy increases the expression of muscarinic and androgen receptors, implying that these receptors are downregulated under physiological conditions and play a role in the better control of reproductive functions under these conditions [84].

Few studies have investigated the effects of castration on the development or consequences of testosterone absence on the MPG in other species, such as cats, dogs, and pigs. Studies in pigs indicate that androgens are also important for neuron survival. Castration significantly affects the survival of pelvic ganglion neurons, with a loss of 74% and 90% after 3 and 6 months, respectively. These conditions also show an increase in caspase 3 expression, suggesting that neurons suffer apoptosis by lack of androgen [17]. Thus, the decrease or increase in certain neurotransmitters or neuropeptides affected by castration may also be a result of testosterone deprivation, enhancing neuronal responses without significant effects when the hormone is reintroduced, at least in boars [69].

## 2. Conclusions

Pelvic functions in various species, including rats, cats, dogs, and pigs, are regulated by the pelvic and hypogastric nerves. The anatomical arrangement may vary slightly, forming either a well-developed ganglion or a plexus. Regardless of the specific structure, the neurons within these systems are regulated and contain two classic neurotransmitters: norepinephrine and acetylcholine. These neurons possess receptors for both neurotransmitters, as well as receptors for testosterone and prolactin, with the latter notably expressed in rats. The presence of testosterone is crucial for the proper functioning of these neurons. Additionally, it is noteworthy that neurons in different species also contain neuropeptides such as VIP, substance P, NPY, SOM, GAL, and CGRP. These neuropeptides can be co-released with neurotransmitters to regulate various pelvic functions, including micturition, defecation, erection, and the control of accessory glands related to reproduction.

## Data Availability

No new data were created or analyzed in this study.
